# Defective T-Cell Apoptosis and T-Regulatory Cell Dysfunction in Rheumatoid Arthritis

**DOI:** 10.3390/cells7120223

**Published:** 2018-11-22

**Authors:** Charles J. Malemud

**Affiliations:** 1 Department of Medicine, Division of Rheumatic Diseases, Case Western Reserve University School of Medicine, Foley Medical Building, 2061 Cornell Road, Suite 207, Cleveland, OH 44122-5076, USA; cjm4@cwru.edu; Tel.: +1-(216)-844-7846 or +1-(216)-536-1945; Fax: +1-(216)-844-2288; 2 Department of Medicine, University Hospitals Cleveland Medical Center, Cleveland, OH 44106, USA

**Keywords:** apoptosis, inflammation, T-lymphocytes, rheumatoid arthritis, synovial fibroblasts

## Abstract

Rheumatoid arthritis (RA) is a chronic, progressive, systemic autoimmune disease that mostly affects small and large synovial joints. At the molecular level, RA is characterized by a profoundly defective innate and adaptive immune response that results in a chronic state of inflammation. Two of the most significant alterations in T-lymphocyte (T-cell) dysfunction in RA is the perpetual activation of T-cells that result in an abnormal proliferation state which also stimulate the proliferation of fibroblasts within the joint synovial tissue. This event results in what we have termed “apoptosis resistance”, which we believe is the leading cause of aberrant cell survival in RA. Finding therapies that will induce apoptosis under these conditions is one of the current goals of drug discovery. Over the past several years, a number of T-cell subsets have been identified. One of these T-cell subsets are the T-regulatory (T_reg_) cells. Under normal conditions T_reg_ cells dictate the state of immune tolerance. However, in RA, the function of T_reg_ cells become compromised resulting in T_reg_ cell dysfunction. It has now been shown that several of the drugs employed in the medical therapy of RA can partially restore T_reg_ cell function, which has also been associated with amelioration of the clinical symptoms of RA.

## 1. Introduction

Rheumatoid arthritis (RA) is a chronic, progressive inflammatory disease of synovial joints [1]. Chronic inflammation results from aberrant innate and adaptive immune responses that help drive RA to the point of joint failure [2,3,4]. Moreover, RA patients have a higher mortality than people in the general population [5] and importantly, the inflammation of RA is not confined to synovial joints [6,7] as manifested by pathologic changes also seen in the cardiovascular and pulmonary system, kidney and skin. In addition, the malfunctioning joints in RA severely limits the patient’s mobility with an associated decline in the quality of life [8].

Several recent reviews have focused on the pathophysiology of RA which emphasized that maladaptive immune responses in RA result from hyperactive T-cell and B-cell patterns of dysfunction [2,9,10,11,12,13]. These abnormalities encompass defective signal transduction [10], and an elevated level of transcription of pro-inflammatory cytokine genes [e.g., TNF-α, IL-1ß, IL-6, IFN-γ] to name only a few [12,14,15]. For example, after T-cells are activated they begin to secrete many of these cytokines. However, abnormal functioning of antigen-presenting cells (APCs) will also frequently accompany autoimmune activation. As such dendritic cells (DCs) acting as APCs also become chronically activated and in doing so function in a maximum antigen-presenting mode which is not their normal state. Therefore, this scenario can result in the imbalances in the patterns of T-cell cytokine secretion that further promotes chronic inflammation. Defective signal transduction also leads to an increased transcription of matrix metalloproteinase (MMP) genes [16]. Thus, MMPs, including MMP-1 (collagenase-1), MMP-2 (72kDa gelatinase), MMP-9 (92kDa gelatinase), MMP-3 (stromelysin-1) and MMP-14 (MT1-MMP) are the activated proteolytic enzymes responsible for the breakdown of extracellular matrix proteins. This component of RA pathology primarily relevant to altered articular cartilage structural integrity is often coupled to an overall impairment of the negative regulators of certain adaptive cellular immune responses [17].

Another facet of RA progression that involves T-cells resides within the two branches of effector CD4^+^ T-cells; the T follicular helper (T_FH_) cells that are programmed to act on B-cells that produce high-affinity class-switched antibodies [18], and the non-T_FH_ effector cells, including the T_h_1, T_h_2 and T_h_17 T-cell subsets. These latter cells exit lymphoid tissues to drive adaptive and innate immune responses [19]. Although the exact operative mechanism that drives this distinction between the types of T-effector cells is not completely understood, a skewing of these T-cell subsets towards one type or the other would be expected to significantly alter the host immune response. In keeping with this, Niu et al. [20] recently suggested that enhanced IL-6/STAT3 signaling could be a mechanism underlying the formation of T_FH_ cells by causing the ratio of T_FH_ cells to T follicular regulator (T_FR_) cells towards the former as RA progresses. Furthermore, Wang et al. [21] confirmed that the ratio of T_FR_ cells to T_FH_ cells decreased as RA progressed and that this skewing of the T_FR_ cells to T_FH_ cells was correlated with changes in serum C-reactive protein, erythrocyte sedimentation rate, rheumatoid factor, anti-cyclic citrullinated peptide antibodies, IgG and the DAS-28, the latter a measure of disease activity.

This narrative review focuses on several aspects of abnormal adaptive cellular and humoral immunity that is characteristic of RA. This would center about some compelling data emerging from recent studies regarding changes occurring in T-cells that may be responsible for “apoptosis-resistance” and would include, the failure of T-cell costimulatory molecules (e.g., cytotoxic T-lymphocyte-associated antigen 4 [CTLA-4]) to regulate T-cell proliferation and the loss of T-regulatory (T_reg_) lymphocyte functions.

In fact, the drug abatacept is a fusion protein that selectively modulates T-cell activation including T-cell proliferation. Thus, abatacept works by binding to CD80 and CD86 receptors on APCs. In that regard, abatacept inhibits T cell activation via this mechanism by selectively interfering with a specific interaction between CD80/CD86 receptors and CD28 thus leading to inhibition of T-cell proliferation and B cell immunological response [22]. In addition, abatacept, which is employed in adult RA and is also approved for the treatment of juvenile idiopathic arthritis was shown to reduce the proliferation of T-helper cells from healthy donors in response to recall antigens as well as reducing the level of IFN-γ and TNF-α in vitro [23]. Of note, the skewing of the T-cell profile towards circulating CCR4^+^CXCR3^−^T-helper subsets, T_h_2 and T_h_17 prior to initiating any RA therapy in patients diagnosed with “early” RA suggested that these cells may have a particular role in the beginning stages of RA [24]. In addition, on the B-cell side, abnormalities in IgG-antigen binding and IgG antigen-forming immune complexes as well as the negative effects of anti-idiotypic antibody production have now been implicated in the progression of RA.

Here we have focused on dysregulated T-cell proliferation and the loss of functional regulation of immune tolerance by T_reg_ cells. These core elements are very critical to adaptive immune cell dysfunction in RA. Therefore it is likely that the attention paid to these abnormalities will further our understanding of how these components contribute to RA pathophysiology. It is also likely to inform us about how these crucial negative regulators of immune cell function become gradually ineffective in the setting of RA. Thus, these changes in immune cell function are mainly responsible for RA progression and promotion of synovial joint articular cartilage degradation and subchondral bone erosions. Furthermore, the attention paid to these abnormalities has resulted in published research related to the extent to which medical therapies for RA could restore control of T-cell proliferation via induction of apoptosis and functional T_reg_ cells. In addition, the continued study of these aspects of RA pathology are proposed in order to stimulate the development of specific novel therapeutics for RA. Importantly, recent findings in this field of research have promoted an upsurge in designing novel drugs to restore T-cell homeostasis.

## 2. T-Cell and RA-Fibroblast-Like Synoviocytes: “Apoptosis-Resistance”

T-cell proliferation is dysregulated in RA. Thus, it was readily predictable that up-regulation of the immune check point proteins, programmed cell death protein 1 ligand 1 (PD-L1) and CTLA-4 would prevent T-cell activation [25] since these proteins represented likely targets for restoring normal T-cell proliferation in RA [26].

Programmed cell death also known as “controlled” cell death or apoptosis is one of the major contributors to maintaining tissue and organ homeostasis [27]. In that regard, RA is characterized, in part, by an imbalance conferred on RA-fibroblast-like synoviocyte (RA-FLS) proliferation by the presence of autoreactive T-cell and B-cell and APCs [28], which render RA-FLS unable to appropriately respond to endogenous mediators that regulate the balance between cell survival and apoptosis, skewing the balance towards cell survival [13]. We have termed this phenomenon “apoptosis resistance” [27,29]. Thus, a combination of events that significantly reduce RA-FLS apoptosis results from the elevated levels of several pro-inflammatory cytokines (e.g., TNF-α, IL-1ß, IL-6, IL-7, IL-8, IL-12/IL-23, IL-15, IL-17, IL-18, IL-32, IFN-γ] and growth factors, including fibroblast growth factor-2 (FGF-2) and vascular endothelial growth factor (VEGF), which cause an aberrant survival of RA-FLS [3,30,31,32,33,34,35]. Many of the downstream events that result in abnormal survival of RA-FLS are dysregulated by constitutive as well as cytokine and growth factor-induced activation of Janus Kinase-Signal Transducers and Activators of Transcription (JAKTAT) [36,37,38,39,40], Stress-Activated Protein Kinase/Mitogen-Activated Protein Kinase (SAPK/MAPK) [40,41,42,43] and PI3K/Akt/Phosphatase and tensin homolog deleted on chromosome 10 (PTEN)/mechanistic target of rapamycin (mTOR) signaling [10,44,45,46,47,48,49] signaling pathways which regulate the balance between cell survival and apoptosis. PTEN is especially important in this context due to the fact that under normal conditions PTEN is a tumor suppressor acting through its phosphatase activity and as such, PTEN regulates cell cycle progression. However, because dysregulated cell proliferation is common in RA, it is likely that abnormalities in PTEN play a critical role in aberrant nonimmune and immune-cell proliferation [49].

Several of the pro-inflammatory cytokines (e.g., TNF-α, IL-1ß) and other molecules (e.g., Fas, VEGF) implicated in the progression of RA activate the extrinsic apoptosis pathway involving the coupling of a protein ligand to a cognate receptor, whereas the intrinsic apoptosis cascade is initiated when cytochrome c is released from mitochondria. This latter event requires energy generated by ATP hydrolysis [50]. In the intrinsic apoptosis pathway, the capacity to release cytochrome c from mitochondria is a function of the balance between the Bcl-2 family of pro-apoptotic and anti-apoptotic proteins [51,52]. Thus, the Bcl-2 protein family, consisting of B-cell lymphoma-2 (Bcl-2), B-cell lymphoma-extra-Large (Bcl-xL), myeloid cell leukemia sequence-1 (Mcl-1), Bacterioides fragilis toxin-1 (Bft-1), Bcl-2 antagonist/killer (Bak), Bcl-2-like protein 11 (Bim), B-cell related ovarian killer (Bok), A1 protein, and B-cell lymphoma-w (Bcl-w) were identified as the main anti-apoptosis proteins whereas Bcl-2-associated x protein (Bax), Bcl-2 homologous antagonist killer (Bak) and B-cell lymphoma-xS (Bcl-xS) were characterized as the major pro-apoptotic proteins. Of these several apoptotic regulators, Bcl-2, Bcl-X, Bcl-xL, Mcl-1, Bim, and Bax have been most thoroughly studied in an attempt to determine their precise role in synovial tissue, myeloid DC and chondrocyte apoptosis [53,54,55,56,57,58]. Of note, the expression of Bim, particularly in macrophages was reduced in RA synovial tissue compared to controls [59], whereas Lee et al. [60] showed that IL-17-mediated Bcl-2 expression was dependent on phospho-STAT3 for promoting the survival of RA-FLS.

Therefore based on our understanding of the various components involved in regulating apoptosis we can envision what the main defects are in the T-cell apoptosis cascade that perpetuates the survival of activated nonimmune and immune cells in the RA milieu. To begin with, the results of several classical studies performed with RA cells had already linked defective Fas-signaling, defective Fas Associated Death Domain (FADD) protein synthesis and/or recruitment, over-activation of nuclear factor-κB (NF-κB), altered Signal Transducers and Activators of Transcription-3 (STAT3) signaling, BH3-only proteins, low expression of p53 [61,62,63], low FADD-like IL-1ß-Converting Enzyme (FLICE) Inhibitory Protein (FLIP) levels [61], over-expression of the anti-apoptosis protein, sentrin [64], aberrant PI3K signaling [49] via PTEN [65] to defective regulation of T-cell and RA-FLS apoptosis.

Additional strategies have been exploited in an attempt to induce apoptosis in RA cells. These modalities included treating RA-FLS with anti-Apo2L/TNF-related apoptosis-inducing ligand (TRAIL), also known as CD253 [66]. Of note, apoptosis could be initiated in normal FLS with recombinant TRAIL (rTRAIL) [67]. However, pretreatment of FLS with IFN-γ blunted rTRAIL-induced apoptosis suggesting that pro-inflammatory cytokines such as IFN-γ which activate JAK-STAT signaling [38] may be involved in suppressing rTRAIL-mediated apoptosis in T-cells and RA-FLS. Despite this finding Neve et al. [68] proposed that TRAIL be tested to determine its effectiveness in promoting apoptosis in synoviocytes and infiltrating lymphocytes collected from RA patients. Furthermore, specifically targeting autoreactive CD8^+^ cells to induce apoptosis in these cell types has also been considered as a preemptive measure designed to reduce the state of chronic inflammation in RA [69].

## 3. T_reg_ Cell Function

T_reg_ cells are a subset of CD4^+^CD25^+^ lymphocytes that express the forkhead box P3 (*Foxp3*) gene and are responsible for maintaining immune tolerance [70]. T_reg_ cells accomplish this function through the release of IL-10 and transforming growth factor-ß (TGF-ß) resulting in immune suppression. T_reg_ cells suppress self-reactive T-cells through cell-to-cell interaction that is mediated by membrane-bound molecules, such as CTLA-4 [71]. Thus, under normal conditions CTLA-4 blocks T-cell activation by binding to CD80/86 more avidly than CD28. Of note, T_reg_ cells expressing Foxp3 can be distinguished from other T-cell subsets such as Tr1, Th3 and CD8^+^CD28^+^, which also exhibit suppressor function.

It has been postulated that the reduced numbers of T_h_17 cells in patients with established RA may have resulted from the shift of T_h_17 cells to either T_h_1 or T_reg_ cells [72]. More recently, Kotake et al. [73] in discussing the plasticity of T_h_17 cells showed that in peripheral blood of early-onset RA patients, the ratio of CD161^+^ T_h_1 was elevated, which correlated with IFN-γ from (INF-γ)^+^T_h_17 cells and was inversely related to anti-CCP antibodies.

Indeed, functional defects in T_reg_ cells have been conjectured to play a critical role in the pathogenesis of RA as well as other autoimmune diseases [74], although the majority of previous studies indicated that T_reg_ cells in RA retained some suppressive activity [75,76]. However, when a functional deficiency in T_reg_ cells was reported, the results of a recent study by Sun et al. [77] traced T_reg_ cell functional deficiency in RA to the reduced expression of T-cell immunoglobulin and mucin-domain containing-3 protein (Tim3). Indeed, the proportion of T_reg_ cells defined by both Foxp3 and CD25^+^ was lower in RA patients than in a control group [78]. However, the proportion of these cells was higher in synovial fluid than in peripheral blood in these RA patients necessitating the need to confirm or refute any differences in T_reg_ cell function between the two compartments. Logically, an extension of these results designed to probe the functionality of T_reg_ cells under various conditions would eventually lead to a spate of recent studies that explored the response of T_reg_ cells to various medical therapies approved for RA. The results of these studies are summarized in Table 1.

Thus, the take-home message from the results of the studies shown in Table 1 is that the number of T_reg_ cells as well as T_reg_ function can be restored with medical therapies that are already approved for RA (e.g., methotrexate, adalimumab, tocilizumab) as well as by tregalizumab, a drug in development for RA. However, study results with abatacept on T_reg_ cell levels were variable with one study indicating a loss of Foxp3-containing cells compared to Ror-γt-containing T-cells [81] whereas another study indicated that abatacept therapy resulted in a rise in T_reg_ cells [82].

Additional recent study results have also illuminated several mechanisms that may be required for the restoration of T_reg_ function in autoimmune arthritis. Thus, Klocke et al. [85] reported that CTLA-4, which contributes to altered T_reg_ function in human RA did not have the same effect on autoreactive T-cells as CTLA-4 had on T_reg_ cells from mice with collagen-induced arthritis (CIA). In the mouse study, the dominant collagen Type-II T-cell epitope was employed to induce arthritis, which was compared to the collagen Type-II epitope mutated at E266D in mouse cartilage. As expected, CTLA-4 expression was required to dampen arthritis severity but only conventional T-cells were required to dampen naïve autoreactive T-cells. However, CTLA-4 expressed on T_reg_ cells prevented inflammation. Taken together the data from this study suggested a “window-of-opportunity” when CTLA-4 expression on T_reg_ cells was likely to be most critical in having an effect tantamount to ameliorating the clinical symptoms of RA.

Another study has identified PTEN as a major contributor to T_reg_ function. Thus, systemic infusion of PTEN to mice with CIA reduced the severity of arthritis while over-expression of PTEN decreased T-cell activation and also differentially modulated T_h_17 and T_reg_ cell function [86]. Of note, in this study, a deficiency in p53 was accompanied by reduced *PTEN* gene expression, which also induced phosphorylation of STAT3 and exacerbated autoimmune arthritis. Therefore, this finding suggested that PTEN could potentially be exploited to modify T_reg_ cell function.

Most recently, Safari et al. [87] reported that the genome editing technology known as Clustered Regularly Interspaced Short Palindromic Repeats (CRISPR) in combination with the CRISPR-associated (Cas) 9 system had the capacity to alter T_reg_ cells. Thus CRISPR-Cas9 could eventually become useful for recruiting T_reg_ cells ex vivo for use in a modality of RA personalized therapy.

## 4. Conclusions and Future Perspectives

The inability of T-cells to undergo apoptosis in response to appropriate signaling molecules, such as IL-1ß, TNF-α and Fas, which are capable of inducing cell death under normal conditions, is a hallmark of RA progression. In that regard, it is now recognized that several molecules involved in RA pathophysiology that should be involved in the induction of apoptosis, including CTLA-4, are not working properly. Thus, survival of activated T-cells ensures that both nonimmune cells such as FLS as well as immune cells, including B-cells, macrophages, DCs, mast cells and neutrophils continue to survive where they promote the chronic inflammatory milieu of RA. Therefore the search must continue to identify appropriate therapeutics that will induce T-cell apoptosis which would likely result in ameliorating the progression of damage to synovial tissue, articular cartilage and subchondral bone that are typically present in the synovial joints of RA patients. Another aspect consistent with this theme is the inability of T_reg_ cells to adequately regulate immune responses in RA. Thus, a targeted approach to restoring aberrant T_reg_ cell function may ameliorate the severity of arthritis when tested in experimental animal models of the disease. Moreover, several drugs that have demonstrated clinical efficacy in RA patients, including methotrexate, adalimumab and tocilizumab result in the production of increased numbers of T_reg_ cells with a concomitant improvement in the functioning of these cells. Newer approaches for editing the genome of T_reg_ cells using CRISPR-Cas 9 technology may eventually provide clinicians with the opportunity to treat RA patients with edited T_reg_ cells for personalized medical therapy.

## Figures and Tables

**Table 1:** The Effect of RA Drug Therapies on T_reg_ Cells.

Drug Therapy & Target	Effect(s) on T_reg_ Cells	Reference
Methotrexate (General Immunosuppressant)	↑ Frequency of CD39^+ 1^ and CD4^+^ CD25^+^ CD39^+ 1^ T_reg_ Cells	[79]
Methotrexate	Restored T_reg_ cell function via demethylation of *FOXP3* locus	[80]
Abatacept (Target: CTLA-4;CD80/86-CD28 Blockade)	↓ Foxp3^+^/Ror-γt ^2^	[81]
Abatacept	↑ T_reg_ cells; Diminished suppression of responder T-cell proliferation in RA	[82]
Tocilizumab (Target: membrane and soluble IL-6R)	↑ Foxp3^+^/Ror-γt ^2^	[81]
Tregalizumab ^3^ (Target: CD39)	Induced T_reg_ Cell Activation	[83]
Adalimumab (Target: TNF-α)	↑ T_reg_ cells in RA patients who responded favorably to treatment	[84]

^1^ CD39 is an ectonucleotidase highly expressed on T_reg_ Cells. ^2^ A transcription factor that characterizes T_h_17 cells; ^3^ humanized CD4-specific monoclonal antibody.
